# 2α,8α-Diacet­oxy-*cis*-himachalane

**DOI:** 10.1107/S160053681003727X

**Published:** 2010-09-25

**Authors:** Ahmed Benharref, Jean Claude Daran, Moha Berraho

**Affiliations:** a Laboratoire de Chimie Biomoléculaires, Substances Naturelles et Réactivité, URAC16,Faculté des Sciences Semlalia, BP 2390 Bd My Abdellah, 40000 Marrakech, Morocco; b Laboratoire de Chimie de Coordination, 205 route de Narbonne, 31077 Toulouse Cedex 04, France

## Abstract

The title compound, C_19_H_32_O_4_, was synthesized from γ-himachalene, wich was isolated from essential oils of *Cedrus atlantica*. The mol­ecule is built up from two fused six- and seven-membered rings. The six-membered ring has a screw-boat conformation, whereas the seven-membered ring displays a half-chair conformation; the dihedral angle between the mean planes of the rings is 61.99 (6)°.

## Related literature

For background to γ-himachalene derivatives, see: Lassaba *et al.* (1998 ▶); Plattier & Teisseire (1974 ▶); Plattier *et al.* (1974 ▶). For a related structure, see: Chiaroni *et al.* (1996 ▶). For ring puckering analysis, see: Cremer & Pople (1975 ▶).
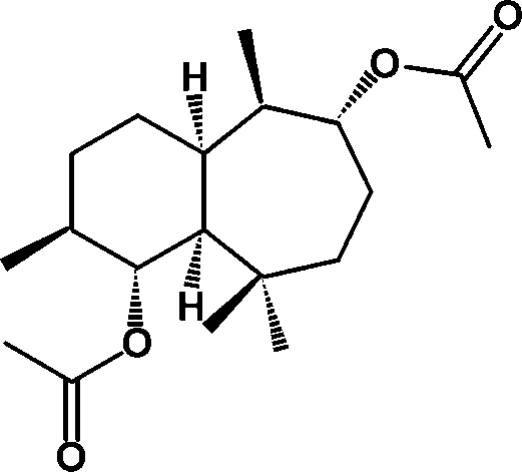


## Experimental

### 

#### Crystal data

C_19_H_32_O_4_
*M* *_r_* = 324.45
Monoclinic, 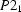

*a* = 5.9316 (2) Å
*b* = 11.7720 (3) Å
*c* = 13.3535 (4) Å
β = 99.603 (3)°
*V* = 919.37 (5) Å^3^
*Z* = 2
Mo *K*α radiation
μ = 0.08 mm^−1^
*T* = 180 K
0.75 × 0.45 × 0.23 mm

#### Data collection

Oxford Diffraction Xcalibur Eos Gemini Ultra diffractometer
Absorption correction: multi-scan (*CrysAlis PRO*; Oxford Diffraction, 2010 ▶) *T*_min_ = 0.799, *T*_max_ = 1.000
3681 measured reflections
1976 independent reflections
1894 reflections with *I* > 2σ(*I*)
*R*_int_ = 0.012

#### Refinement

*R*[*F* ^2^ > 2σ(*F* ^2^)] = 0.030
*wR*(*F* ^2^) = 0.079
*S* = 1.06
1976 reflections
214 parameters
1 restraint
H-atom parameters constrained
Δρ_max_ = 0.17 e Å^−3^
Δρ_min_ = −0.14 e Å^−3^

### 

Data collection: *CrysAlis PRO* (Oxford Diffraction, 2010 ▶); cell refinement: *CrysAlis PRO*; data reduction: *CrysAlis PRO*; program(s) used to solve structure: *SIR2004* (Burla *et al.*, 2005 ▶); program(s) used to refine structure: *SHELXL97* (Sheldrick, 2008 ▶); molecular graphics: *ORTEP-3 for Windows* (Farrugia, 1997 ▶); software used to prepare material for publication: *WinGX* (Farrugia, 1999 ▶).

## Supplementary Material

Crystal structure: contains datablocks I, global. DOI: 10.1107/S160053681003727X/im2232sup1.cif

Structure factors: contains datablocks I. DOI: 10.1107/S160053681003727X/im2232Isup2.hkl

Additional supplementary materials:  crystallographic information; 3D view; checkCIF report

## Figures and Tables

**Fig. 1.**
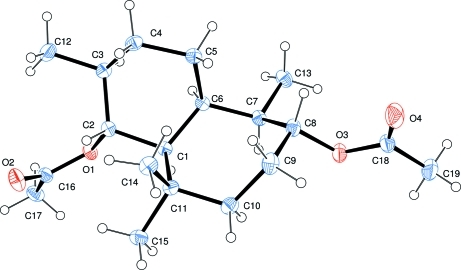
: Molecular structure of the title compound with the atom-labelling scheme. Displacement ellipsoids are drawn at the 30% probability level. H atoms are represented as small spheres of arbitrary radii.

## supplementary crystallographic information

### Comment

This work is a part of our ongoing program concerning the valorization of the most abundant essential oils in Morocco, such as *Cedrus atlantica*. This oil is made up mainly (75%) of bicyclic sesquiterpene hydrocarbons, among which is found the compound γ- himachalene (Plattier & Teisseire, 1974). This compound is a minority product of the mixture of hydrocarbons of essential oil of the Atlas Cedar (*Cedrus atlantica*), isolated by Plattier *et al.* (1974). Literature provides only a few articles on the reactivity of this sesquiterpene, namely its hydrochlorination (Plattier & Teisseire, 1974), its hydroboration (Plattier *et al.*, 1974) and its epoxidation (Chiaroni *et al.*,1996; Lassaba *et al.*, 1998). Thus the action of two equivalents of diborane followed by oxidation with hydrogen peroxide and soda leads to two diasterioisomers: *cis*-himachal-2α,8α-diol and *cis*-himachal-2α,8β-diol. In order to prepare products with high added value, we have treated the mixture of these two isomers with the acetic anhydride in pyridine (see experimental) and obtained a mixture of two isomers (*X*) and (Y) with a combined yield of 98%. A study of spectral analysis of ^1^H NMR, ^13^C NMR and mass spectrometry did not allow us to differentiate between the structures of both isomers. Nevertheless, a X-ray crystallographic study of a single-crystal of (*X*) has allowed its identification as 2α,8α-diacetoxy-*cis*-himachalane (Scheme 1) and distinguish it from its isomer (Y), which is 2α,8β-diacetoxy-*cis*-himachalane. The molecule (Fig.1) is built up from two fused six- and seven-membered rings with the acetoxy groups at positions 2 and 8 in α-configuration. The six-membered ring has a screw boat conformation as indicated by the total puckering amplitude QT = 0.7585 (12) Å and a spherical polar angle of θ= 92.06 (9)° with φ= 71.42 (9)°. The seven-membered ring displays a half chair conformation with QT =0.8062 (13) Å, θ = 42.17 (9)°, φ2 = -26.97 (14)° and φ3 = 169.95 (13)° (Cremer & Pople, 1975).

### Experimental

To a solution of 1 g of the mixture of two isomers of *cis*-himachal-2,8-diol (prepared from γ-himachalene) in 30 ml of pyridine was added 20 ml of acetic anhydride. The mixture is stirred overnight at room temperature, then treated with 100 ml of ice water. The reaction mixture was extracted three times with 30 ml of ether. The organic phases obtained are dried over sodium sulfate and then concentrated under vacuum. The residue obtained is chromatographed on silica gel column impregnated with silver nitrate (10%) with a mixture of hexane - ethyl acetate (95–5) used as eluent. The two diastereoisomers 2α,8α-diacetoxy-*cis*-himachalane (*X*) and 2α,8β-diacetoxy-*cis*-himachalane (Y) are obtained by this procedure in a 80/20 ratio and a combined yield of 98%. The title compound (isomer *X*) is recrystallized from a mixture of hexane and ethyle acetate (40/60).

### Refinement

All H atoms were fixed geometrically and treated as riding with C—H = 0.96 Å (methyl), 0.97 Å (methylene) and 0.98 Å (methine) with *U*_iso_(H) = 1.2 *U*_eq_(CH_2_, CH) or *U*_iso_(H) = 1.5 *U*_eq_(CH_3_). In the absence of significant anomalous scattering, the absolute configuration could not be reliably determined and thus 1705 Friedel pairs were merged and any references to the Flack parameter were removed.

### Crystal data

**Table:**

C_19_H_32_O_4_	*F*(000) = 356
*M**_r_* = 324.45	*D*_x_ = 1.172 Mg m^−^^3^
Monoclinic, *P*2_1_	Mo *K*α radiation, λ = 0.71073 Å
Hall symbol: P 2yb	Cell parameters from 8181 reflections
*a* = 5.9316 (2) Å	θ = 3.5–29.2°
*b* = 11.7720 (3) Å	µ = 0.08 mm^−^^1^
*c* = 13.3535 (4) Å	*T* = 180 K
β = 99.603 (3)°	Box, colourless
*V* = 919.37 (5) Å^3^	0.75 × 0.45 × 0.23 mm
*Z* = 2	

### Data collection

**Table:**

Oxford Diffraction Xcalibur Eos Gemini Ultra diffractometer	1976 independent reflections
Radiation source: fine-focus sealed tube	1894 reflections with *I* > 2σ(*I*)
graphite	*R*_int_ = 0.012
Detector resolution: 16.1978 pixels mm^-1^	θ_max_ = 26.4°, θ_min_ = 3.5°
ω scans	*h* = −7→7
Absorption correction: multi-scan (*CrysAlis PRO*; Oxford Diffraction, 2010)	*k* = −14→14
*T*_min_ = 0.799, *T*_max_ = 1.000	*l* = 0→16
3681 measured reflections	

### Refinement

**Table:**

Refinement on *F*^2^	Primary atom site location: structure-invariant direct methods
Least-squares matrix: full	Secondary atom site location: difference Fourier map
*R*[*F*^2^ > 2σ(*F*^2^)] = 0.030	Hydrogen site location: inferred from neighbouring sites
*wR*(*F*^2^) = 0.079	H-atom parameters constrained
*S* = 1.06	*w* = 1/[σ^2^(*F*_o_^2^) + (0.0489*P*)^2^ + 0.0959*P*] where *P* = (*F*_o_^2^ + 2*F*_c_^2^)/3
1976 reflections	(Δ/σ)_max_ < 0.001
214 parameters	Δρ_max_ = 0.17 e Å^−^^3^
1 restraint	Δρ_min_ = −0.13 e Å^−^^3^

### Special details

**Table:**

Experimental. Empirical absorption correction using spherical harmonics, implemented in SCALE3 ABSPACK scaling algorithm. CrysAlisPro (Oxford Diffraction 2010)
Geometry. All e.s.d.'s (except the e.s.d. in the dihedral angle between two l.s. planes) are estimated using the full covariance matrix. The cell e.s.d.'s are taken into account individually in the estimation of e.s.d.'s in distances, angles and torsion angles; correlations between e.s.d.'s in cell parameters are only used when they are defined by crystal symmetry. An approximate (isotropic) treatment of cell e.s.d.'s is used for estimating e.s.d.'s involving l.s. planes.
Refinement. Refinement of *F*^2^ against ALL reflections. The weighted *R*-factor *wR* and goodness of fit *S* are based on *F*^2^, conventional *R*-factors *R* are based on *F*, with *F* set to zero for negative *F*^2^. The threshold expression of *F*^2^ > σ(*F*^2^) is used only for calculating *R*-factors(gt) *etc*. and is not relevant to the choice of reflections for refinement. *R*-factors based on *F*^2^ are statistically about twice as large as those based on *F*, and *R*- factors based on ALL data will be even larger.

### Fractional atomic coordinates and isotropic or equivalent isotropic displacement parameters (Å^2^)

**Table:**

	*x*	*y*	*z*	*U*_iso_*/*U*_eq_	
C1	0.0099 (3)	0.89742 (14)	0.20021 (12)	0.0220 (3)	
H1	0.1630	0.8831	0.1845	0.026*	
C2	−0.1412 (3)	0.92089 (14)	0.09491 (12)	0.0245 (3)	
H2	−0.2622	0.8634	0.0827	0.029*	
C3	−0.2509 (3)	1.03856 (15)	0.08423 (12)	0.0270 (4)	
H3	−0.1305	1.0954	0.0833	0.032*	
C4	−0.3655 (3)	1.05982 (16)	0.17728 (13)	0.0286 (4)	
H4A	−0.4791	1.0013	0.1805	0.034*	
H4B	−0.4441	1.1323	0.1694	0.034*	
C5	−0.1940 (3)	1.06035 (16)	0.27780 (12)	0.0271 (4)	
H5A	−0.1699	1.1378	0.3020	0.033*	
H5B	−0.2567	1.0173	0.3287	0.033*	
C6	0.0352 (3)	1.00838 (14)	0.26315 (12)	0.0226 (3)	
H6	0.0943	1.0628	0.2183	0.027*	
C7	0.2205 (3)	1.00777 (15)	0.35903 (12)	0.0254 (3)	
H7	0.3489	0.9630	0.3427	0.030*	
C8	0.1503 (3)	0.95438 (17)	0.45333 (12)	0.0289 (4)	
H8	0.0556	1.0087	0.4834	0.035*	
C9	0.0274 (3)	0.84093 (19)	0.44034 (13)	0.0354 (4)	
H9A	−0.1359	0.8555	0.4284	0.042*	
H9B	0.0630	0.7996	0.5038	0.042*	
C10	0.0816 (3)	0.76424 (16)	0.35535 (13)	0.0311 (4)	
H10A	0.0590	0.6861	0.3744	0.037*	
H10B	0.2424	0.7731	0.3512	0.037*	
C11	−0.0575 (3)	0.78409 (15)	0.24849 (13)	0.0253 (3)	
C12	−0.4260 (3)	1.05045 (19)	−0.01273 (14)	0.0386 (4)	
H12A	−0.3562	1.0307	−0.0703	0.058*	
H12B	−0.4794	1.1275	−0.0195	0.058*	
H12C	−0.5527	1.0006	−0.0095	0.058*	
C13	0.3102 (3)	1.12841 (17)	0.38390 (15)	0.0341 (4)	
H13	0.1879	1.1755	0.3989	0.051*	
H13A	0.3684	1.1590	0.3267	0.051*	
B	0.4303	1.1262	0.4417	0.051*	
C14	−0.3141 (3)	0.77332 (17)	0.25420 (14)	0.0307 (4)	
H14A	−0.4009	0.7725	0.1867	0.046*	
H14B	−0.3609	0.8367	0.2911	0.046*	
H14C	−0.3404	0.7040	0.2883	0.046*	
C15	0.0020 (3)	0.68616 (16)	0.18139 (14)	0.0331 (4)	
H15A	−0.0356	0.6151	0.2097	0.050*	
H15B	0.1625	0.6880	0.1784	0.050*	
H15C	−0.0838	0.6942	0.1142	0.050*	
C16	−0.0647 (3)	0.84124 (16)	−0.06239 (13)	0.0300 (4)	
C17	0.1060 (4)	0.84018 (18)	−0.13319 (14)	0.0380 (4)	
H17A	0.2502	0.8131	−0.0978	0.057*	
H17B	0.1242	0.9158	−0.1576	0.057*	
H17C	0.0531	0.7910	−0.1896	0.057*	
C18	0.3634 (3)	0.95040 (18)	0.62200 (13)	0.0362 (4)	
C19	0.5969 (4)	0.9380 (2)	0.68344 (16)	0.0516 (6)	
H19A	0.5892	0.8902	0.7410	0.077*	
H19B	0.6542	1.0114	0.7064	0.077*	
H19C	0.6974	0.9044	0.6424	0.077*	
O1	0.0055 (2)	0.90900 (10)	0.01763 (9)	0.0275 (3)	
O2	−0.2420 (3)	0.78940 (14)	−0.07569 (10)	0.0456 (4)	
O3	0.3679 (2)	0.93985 (13)	0.52241 (9)	0.0338 (3)	
O4	0.1927 (3)	0.96866 (19)	0.65590 (11)	0.0572 (5)	

### Atomic displacement parameters (Å^2^)

**Table:**

	*U*^11^	*U*^22^	*U*^33^	*U*^12^	*U*^13^	*U*^23^
C1	0.0175 (7)	0.0237 (8)	0.0255 (7)	−0.0015 (6)	0.0060 (6)	−0.0003 (7)
C2	0.0232 (7)	0.0266 (9)	0.0246 (8)	−0.0040 (6)	0.0071 (6)	−0.0009 (6)
C3	0.0260 (8)	0.0290 (9)	0.0267 (8)	0.0013 (7)	0.0066 (6)	0.0031 (7)
C4	0.0235 (8)	0.0306 (9)	0.0330 (9)	0.0040 (7)	0.0084 (6)	0.0037 (8)
C5	0.0252 (8)	0.0301 (9)	0.0280 (8)	0.0020 (7)	0.0100 (6)	−0.0012 (7)
C6	0.0217 (7)	0.0240 (8)	0.0236 (7)	−0.0017 (6)	0.0081 (6)	0.0001 (6)
C7	0.0220 (8)	0.0302 (9)	0.0250 (8)	−0.0013 (7)	0.0073 (6)	−0.0035 (7)
C8	0.0249 (8)	0.0396 (10)	0.0230 (8)	0.0014 (8)	0.0060 (6)	−0.0020 (8)
C9	0.0334 (9)	0.0472 (11)	0.0267 (9)	−0.0070 (9)	0.0089 (7)	0.0054 (9)
C10	0.0305 (8)	0.0294 (9)	0.0333 (9)	−0.0020 (7)	0.0052 (7)	0.0067 (8)
C11	0.0228 (7)	0.0239 (8)	0.0293 (8)	−0.0013 (7)	0.0050 (6)	0.0015 (7)
C12	0.0380 (10)	0.0430 (11)	0.0326 (9)	0.0067 (9)	−0.0009 (7)	0.0047 (9)
C13	0.0315 (9)	0.0360 (10)	0.0360 (10)	−0.0064 (8)	0.0092 (7)	−0.0082 (8)
C14	0.0253 (8)	0.0303 (9)	0.0371 (9)	−0.0061 (7)	0.0066 (7)	0.0048 (8)
C15	0.0358 (9)	0.0248 (9)	0.0388 (10)	−0.0002 (7)	0.0066 (8)	−0.0002 (8)
C16	0.0406 (9)	0.0248 (8)	0.0236 (8)	−0.0001 (8)	0.0027 (7)	0.0002 (7)
C17	0.0534 (12)	0.0319 (10)	0.0309 (9)	0.0023 (9)	0.0133 (8)	−0.0034 (8)
C18	0.0507 (11)	0.0320 (10)	0.0248 (8)	−0.0009 (9)	0.0034 (8)	−0.0017 (8)
C19	0.0615 (14)	0.0486 (13)	0.0372 (11)	−0.0010 (11)	−0.0137 (9)	−0.0020 (10)
O1	0.0289 (6)	0.0309 (7)	0.0242 (6)	−0.0041 (5)	0.0086 (5)	−0.0040 (5)
O2	0.0528 (8)	0.0469 (9)	0.0364 (7)	−0.0179 (7)	0.0053 (6)	−0.0092 (7)
O3	0.0298 (6)	0.0464 (8)	0.0246 (6)	0.0022 (6)	0.0024 (5)	−0.0034 (6)
O4	0.0629 (10)	0.0831 (13)	0.0278 (7)	0.0126 (10)	0.0140 (7)	−0.0011 (8)

### Geometric parameters (Å, °)

**Table:**

C1—C6	1.547 (2)	C10—H10A	0.9700
C1—C2	1.561 (2)	C10—H10B	0.9700
C1—C11	1.562 (2)	C11—C15	1.537 (3)
C1—H1	0.9800	C11—C14	1.542 (2)
C2—O1	1.4632 (19)	C12—H12A	0.9600
C2—C3	1.527 (2)	C12—H12B	0.9600
C2—H2	0.9800	C12—H12C	0.9600
C3—C12	1.525 (2)	C13—H13	0.9600
C3—C4	1.533 (2)	C13—H13A	0.9600
C3—H3	0.9800	C13—B	0.9600
C4—C5	1.543 (2)	C14—H14A	0.9600
C4—H4A	0.9700	C14—H14B	0.9600
C4—H4B	0.9700	C14—H14C	0.9600
C5—C6	1.533 (2)	C15—H15A	0.9600
C5—H5A	0.9700	C15—H15B	0.9600
C5—H5B	0.9700	C15—H15C	0.9600
C6—C7	1.542 (2)	C16—O2	1.203 (2)
C6—H6	0.9800	C16—O1	1.343 (2)
C7—C8	1.526 (2)	C16—C17	1.496 (3)
C7—C13	1.533 (3)	C17—H17A	0.9600
C7—H7	0.9800	C17—H17B	0.9600
C8—O3	1.466 (2)	C17—H17C	0.9600
C8—C9	1.518 (3)	C18—O4	1.195 (2)
C8—H8	0.9800	C18—O3	1.340 (2)
C9—C10	1.526 (3)	C18—C19	1.495 (3)
C9—H9A	0.9700	C19—H19A	0.9600
C9—H9B	0.9700	C19—H19B	0.9600
C10—C11	1.542 (2)	C19—H19C	0.9600
			
C6—C1—C2	109.21 (13)	C9—C10—H10A	108.1
C6—C1—C11	120.34 (13)	C11—C10—H10A	108.1
C2—C1—C11	112.02 (13)	C9—C10—H10B	108.1
C6—C1—H1	104.6	C11—C10—H10B	108.1
C2—C1—H1	104.6	H10A—C10—H10B	107.3
C11—C1—H1	104.6	C15—C11—C14	106.96 (14)
O1—C2—C3	108.37 (13)	C15—C11—C10	106.64 (14)
O1—C2—C1	107.38 (12)	C14—C11—C10	108.78 (14)
C3—C2—C1	114.61 (14)	C15—C11—C1	107.49 (13)
O1—C2—H2	108.8	C14—C11—C1	114.41 (14)
C3—C2—H2	108.8	C10—C11—C1	112.14 (14)
C1—C2—H2	108.8	C3—C12—H12A	109.5
C12—C3—C2	112.44 (15)	C3—C12—H12B	109.5
C12—C3—C4	109.97 (14)	H12A—C12—H12B	109.5
C2—C3—C4	108.18 (14)	C3—C12—H12C	109.5
C12—C3—H3	108.7	H12A—C12—H12C	109.5
C2—C3—H3	108.7	H12B—C12—H12C	109.5
C4—C3—H3	108.7	C7—C13—H13	109.5
C3—C4—C5	112.86 (13)	C7—C13—H13A	109.5
C3—C4—H4A	109.0	H13—C13—H13A	109.5
C5—C4—H4A	109.0	C7—C13—B	109.5
C3—C4—H4B	109.0	H13—C13—B	109.5
C5—C4—H4B	109.0	H13A—C13—B	109.5
H4A—C4—H4B	107.8	C11—C14—H14A	109.5
C6—C5—C4	110.94 (13)	C11—C14—H14B	109.5
C6—C5—H5A	109.5	H14A—C14—H14B	109.5
C4—C5—H5A	109.5	C11—C14—H14C	109.5
C6—C5—H5B	109.5	H14A—C14—H14C	109.5
C4—C5—H5B	109.5	H14B—C14—H14C	109.5
H5A—C5—H5B	108.0	C11—C15—H15A	109.5
C5—C6—C7	114.85 (13)	C11—C15—H15B	109.5
C5—C6—C1	113.48 (13)	H15A—C15—H15B	109.5
C7—C6—C1	116.00 (13)	C11—C15—H15C	109.5
C5—C6—H6	103.4	H15A—C15—H15C	109.5
C7—C6—H6	103.4	H15B—C15—H15C	109.5
C1—C6—H6	103.4	O2—C16—O1	124.46 (16)
C8—C7—C13	109.58 (15)	O2—C16—C17	124.71 (17)
C8—C7—C6	115.63 (13)	O1—C16—C17	110.82 (15)
C13—C7—C6	110.43 (14)	C16—C17—H17A	109.5
C8—C7—H7	106.9	C16—C17—H17B	109.5
C13—C7—H7	106.9	H17A—C17—H17B	109.5
C6—C7—H7	106.9	C16—C17—H17C	109.5
O3—C8—C9	108.97 (15)	H17A—C17—H17C	109.5
O3—C8—C7	103.58 (13)	H17B—C17—H17C	109.5
C9—C8—C7	117.39 (14)	O4—C18—O3	123.42 (18)
O3—C8—H8	108.9	O4—C18—C19	125.14 (18)
C9—C8—H8	108.9	O3—C18—C19	111.43 (18)
C7—C8—H8	108.9	C18—C19—H19A	109.5
C8—C9—C10	116.66 (15)	C18—C19—H19B	109.5
C8—C9—H9A	108.1	H19A—C19—H19B	109.5
C10—C9—H9A	108.1	C18—C19—H19C	109.5
C8—C9—H9B	108.1	H19A—C19—H19C	109.5
C10—C9—H9B	108.1	H19B—C19—H19C	109.5
H9A—C9—H9B	107.3	C16—O1—C2	118.45 (13)
C9—C10—C11	116.86 (15)	C18—O3—C8	116.89 (14)
